# Gel-Forming of Self-Assembling Peptides Functionalized with Food Bioactive Motifs Modulate DPP-IV and ACE Inhibitory Activity in Human Intestinal Caco-2 Cells

**DOI:** 10.3390/biomedicines10020330

**Published:** 2022-01-31

**Authors:** Raffaele Pugliese, Martina Bartolomei, Carlotta Bollati, Giovanna Boschin, Anna Arnoldi, Carmen Lammi

**Affiliations:** 1 NeMO Lab, ASST Grande Ospedale Metropolitano Niguarda, 20162 Milan, Italy; 2 Department of Pharmaceutical Sciences, University of Milan, 20133 Milan, Italy; martina.bartolomei@unimi.it (M.B.); carlotta.bollati@unimi.it (C.B.); giovanna.boschin@unimi.it (G.B.); anna.arnoldi@unimi.it (A.A.)

**Keywords:** self-assembling peptides, co-assembly, supramolecular hydrogels, nano-nutraceuticals, dipeptidyl peptidase IV, angiotensin converting enzyme, bioactivity, mechanical properties

## Abstract

Food bioactive peptides are increasingly used for formulating food products, nutraceuticals, and functional food, since they are generally considered safe for human consumption and metabolic syndrome prevention. They are also becoming popular as sustainable sources of novel functional biomaterials such as hydrogels, edible nanonutraceuticals, delivery systems, and packing materials. However, such food peptides are mostly unstable, and degrade during food processing, or in a gastrointestinal environment, thus resulting in low bioavailability precluding their practical applications. Here, we decided to functionalize the well-known and characterized self-assembling peptide RADA16 with two synthetic analogues of food bioactive peptides deriving from the hydrolysis of soybean glycinin and lupin β-conglutin (namely IAVPTGVA and LTFPGSAED) for control of and improvement in their gel-forming nanostructures, biomechanics, and biological features. Extensive characterization was performed via Circular Dichroism (CD) spectroscopy, Fourier Transform Infrared spectroscopy (FT-IR), Thioflavin T (ThT) binding assay, rheological measurements, and Atomic Force Microscopy (AFM) analysis. Lastly, since self-assembling peptides (SAPs) can be co-assembled with diluent SAPs (without a bioactive epitope) as an approach to control the density of biological signals and therefore attain enhanced bioactivity, we investigated the effect of the co-assembly of RADA16 and functionalized food bioactive SAPs (dubbed cAP-Soy1 and cAP-Lup1) for the growth of Caco-2 human intestinal cells and contextually we characterized their biological activities as DPP-IV and ACE inhibitors, in order to demonstrate their potential use for the prevention of metabolic syndrome.

## 1. Introduction

IAVPTGVA and LTFPGSAED (dubbed, respectively, Soy1 and Lup1) are two peptides derived from the hydrolysis of soybean glycinin and lupin β-conglutin, respectively. Both peptides display multifunctional behavior, since they demonstrated to act as in vitro HMGCoAR and DPP-IV inhibitors, respectively [1,2,3,4]. On human HepG2 cells, through the inhibition of HMGCoAR activity, they can modulate the cholesterol metabolism pathway leading to the upregulation of the LDLR protein levels, thus improved the ability of hepatic cells to uptake LDL from the extracellular environment with an in vitro cholesterol-lowering activity [3,5]. In addition, both Soy1 and Lup1 drop the in vitro and in situ human Caco-2 cells DPP-IV activity [1]. 

However, such food-derived peptides show low stability, bioavailability (due to their rapid degradation by active peptidases located on the intestinal microvilli), and a poor propensity to form functional biomaterials (i.e., hydrogels), thus precluding their practical applications. As we previously reported, one strategy to deal with these issues might be the use of well-designed and controlled self-assembled supramolecular systems, such as self-assembling peptides (SAPs) to be combined with the bioactive peptides in order to create hybrid systems, in which monomers are non-covalently bonded [6,7,8,9]. Indeed, when Soy1 and Lup1 were non-covalently entrapped into the RADA16 self-assembling peptide, the stability of both peptides was significantly improved as well as their capability of inhibiting DPP-IV and ACE, suggesting that this strategy could be a viable platform for targeting metabolic diseases [10].

Since SAPs are amenable to multi-functionalizations with bioactive motifs to intimately interact with cells, cytokines, tissues, and so on; we decided to functionalize the well-known and characterized RADA16 SAP with the synthetic analogues of Soy1 and Lup1 at the N-terminus, in order to create a food bioactive assembling peptide (dubbed fAP) with improved biological properties. 

Extending the peptide sequence to the C or N-terminus, either during the peptide synthesis or subsequently using click-chemistry, the biofunctionalization of the SAPs can be obtained. Furthermore, differently functionalized SAPs, sharing the same self-assembling sequence, can be mixed together, thus displaying multiple functional motifs [11]. Using this simple strategy, it is feasible to customize, in terms of bioactivity, an SAP-based hydrogel for each new biomedical application.

**Figure 1:**
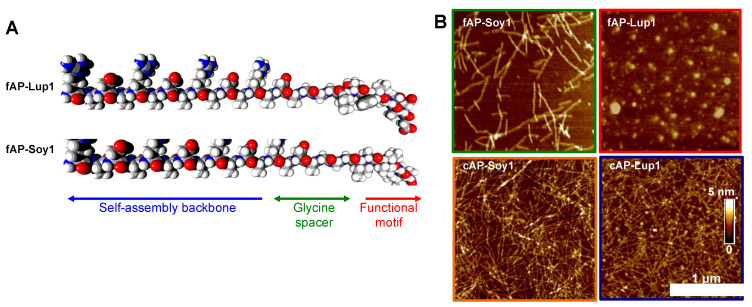
(**A**) Molecular graphics representation of fAP-Lup1 and fAP-Soy1 sequences. (**B**) Atomic force microscopy images of fAP-Soy1, fAP-Lup1, and co-assembled fAPs with a pure RADA16.

Herein, by using the advantage of SAP biofunctionalization, we synthetized two fAP: Ac-IAVPTGVAGGGRADARADARADARADA-CONH_2_ (dubbed fAP-Soy1) and Ac-LTFPGSAEDGGGRADARADARADARADA-CONH_2_ (dubbed fAP-Lup1) (Figure 1A). The N-terminus of both peptides was acetylated to increase their stability when in solution [12]; instead, a three-glycines spacer was used to avoid interference between the food bioactive motifs and the self-assembling backbone [13]. The secondary structures, biomechanics, and nanostructure morphologies of fAP hydrogels were extensively characterized by Fourier transform infrared spectroscopy (FT-IR), circular dichroism (CD), Thioflavin T (ThT) spectroscopy assay, oscillatory stress rheology, and atomic force microscopy (AFM). Lastly, since SAPs can be co-assembled with diluent SAPs (without a bioactive epitope) as an approach to control the density of biological signals [14] and therefore attain enhanced bioactivity in cases where steric effects play an important role in signaling [15], we investigated the effect of the co-assembly of RADA16 and fAPs (dubbed cAP-Soy1 and cAP-Lup1) for the growth of Caco-2 human intestinal cells and contextually we characterized their biological activities as DPP-IV and ACE inhibitors, in order to demonstrate their potential use for the prevention of metabolic syndrome.

This study may facilitate the development of naturally occurring food peptides as functional biomaterials with improved stability, bioavailability, and broaden their applications for metabolic syndrome prevention, food chemistry, coating for packaging, and beyond.

## 2. Materials and Methods

### 2.1. Chemicals

Dulbecco’s modified Eagle’s medium (DMEM), fetal bovine serum (FBS), phosphate buffered saline (PBS), penicillin/streptomycin, and 96-well plates were purchased from Euroclone (Milan, Italy). Gly-Pro-amido-4-methylcoumarin hydrobromide (Gly-Pro-AMC) and MTT [3-(4,5-dimethylthiazol-2-yl)-2,5-diphenyltetrazolium bromide] were from Sigma-Aldrich (St. Louis, MO, USA). Angiotensin I Converting Enzyme Activity (ACE1) Assay Kit came from BioVision (Milpitas, CA, USA).

### 2.2. Peptide Preparation

The synthetic analogues peptides were purchased from GenScript Biotech Corporation (Piscataway, NJ, USA). The purity of lyophilized peptides (>95%) was tested using binary HPLC and LCMS mass spectrometry (Agilent 6520, Santa Clara, CA, USA) (Figure S1). Lyophilized peptides were dissolved at 1% (w/v) in distilled water (GIBCO^®^ Thermo Fisher Scientific, Waltham, MA, USA), and then stored at +4 °C overnight before use. For hydrogel preparation, 1X Dulbecco’s Phosphate Buffered Saline (DPBS) (Ca^2+^/Mg^2+^-free) was used, and the peptide solution was vigorously stirred for 5 min. 

### 2.3. Thioflavin T (ThT) Spectroscopy Assay

ThT analysis of peptides was performed to assess the presence of amyloidogenic fibril structures. Peptide samples (40 μM) were mixed with the ThT solution (20 μM) and stirred for 4 min, as previously reported [16]. The fluorescence intensity of ThT was checked through a Synergy plate reader (Biotek, Bad Friedrichshall, Germany) using λ_ex_ = 440 nm (5 nm bandpass) and λ_em_ = 482 nm (10 nm bandpass), over 60 s at 25 °C. Measurements were normalized over ThT-alone fluorescence and processed with OriginLab^TM^ 8 software.

### 2.4. Fourier Transform Infrared Spectroscopy (FT-IR)

The FT-IR spectra of peptides were obtained using a PerkinElmer Spectrum 100 spectrometer. Twenty acquisitions were recorded for each spectrum; the scan conditions were as follows: 4 cm^−1^ spectrum resolution, 25 kHz scan speed, 1000-scan coaddition, and triangular apodization. All obtained spectra were reported after ATR correction, smoothing, and automatic baseline correction using OriginLab^TM^ 8 software.

### 2.5. Circular Dichroism Spectroscopy (CD)

The CD spectra (180–300 nm) of peptides were recorded on a Jasco J-815 (Jasco Corp., Tokyo, Japan) spectropolarimeter using a 0.1 mm quartz cuvette, as previously reported [17]. All scans were carried out with a scan speed of 50 nm/min, a bandwidth of 1 nm, and time-response of 2 s. A reference spectrum of distilled water was recorded and subtracted from each spectrum. All obtained spectra were reported using OriginLab^TM^ 8 software.

### 2.6. Mechanical Testing

To evaluate the storage (G’) and loss (G’’) moduli of assembled peptides (1% w/v), frequency sweep experiments were recorded as a function of angular frequency (0.1–100 Hz) at a fixed strain of 1%, by using a stress/rate-controlled Rheometer (TA Instruments) equipped with a parallel plate geometry (acrylic diameter 20 mm; gap 34 μm).

### 2.7. Atomic Force Microscopy (AFM)

AFM measurements were captured in tapping mode using a Tosca system (Anton Paar, Graz, Austria) using single-beam silicon cantilever probes. Peptides were dissolved in distilled water at a final concentration of 0.01% w/v and sonicated for 30 min, the same day as imaging. Then, 5 μL of peptide solution were deposited for 5 min on freshly cleaved mica, and distilled water was used to remove peptides loosely bound to the mica.

### 2.8. Cell Culture 

Caco-2 cells (INSERM, Paris, France), were sub-cultured at 50% density (37 °C, 90% air, 10% CO_2_ atmosphere) in Dulbecco Minimum Essential Medium (DMEM) containing 25 mM glucose, 3.7 g/L NaHCO_3_, 4 mM stable L-glutamine, 1% nonessential amino acids, 100 U/L penicillin, 100 µg/L streptomycin (complete medium), supplemented with 10% heat-inactivated fetal bovine serum (FBS Hyclone Laboratories, Logan, UT, USA).

### 2.9. In Situ DPPIV Activity Assay

For 2D cell culture Caco-2 cells were seeded on the surface of cAP-Soy1 and cAP-Lup1 (10 and 100 μM) hydrogels at the density of 5 × 10^4^/well in a black 96-well plate with a clear bottom. The day after, the spent media was removed, cells were washed with 100 µL of DPBS (Ca^2+^/Mg^2+^ free), and 50 µL of DPPIV substrate at the concentration of 50.0 µM in DPBS (Ca^2+^/Mg^2+^ free) were added in each well. Fluorescence signals using λ_ex_ = 350 nm and λ_em_ = 450 nm were measured using the Synergy H1 from Biotek (Bad Friedrichshall, Germany) every 1 min over 10 min. 

### 2.10. In Situ ACE Activity Assay 

For the experiments, Caco-2 cells at the density of 5 × 10^4^ cells/well were seeded on cAP-Soy1 and cAP-Lup1 (10 and 100 μM) hydrogels in a 96-well plate and incubated for 24 h at 37 °C. The following day, the spent media was removed and 2D cell cultures were scraped in 50 μL/well of ice-cold ACE1 lysis buffer and centrifuged at 4 °C, 13,300× *g*, for 15 min, and the supernatant was recovered and transferred into a new ice-cold tube. After quantifying the proteins using the Bradford method, 2 μg of total protein (the equivalent of 2 μL) were added to 18 μL of ACE1 lysis buffer in each well. Subsequently, 20 μL of 4% ACE1 substrate (in assay buffer) were added and the fluorescence was measured in kinetic mode over 10 min at 37 °C using λ_ex_ = 330 nm and λ_em_ = 430. As a background control, 20 µL of ACE1 lysis buffer in 20 µL of ACE1 assay buffer were used. 

### 2.11. MTT Assay

To assess the viability of Caco-2 cells seeded on the cAP-Soy1 and cAP-Lup1 (100 μM) hydrogels, 3-(4,5-dimethylthiazol-2-yl)-2,5-diphenyltetrazolium bromide (MTT) assay was used. After 72 h of cell seeding, cells were incubated with 100 µL/well of MTT reagent for 2 h at 37 °C in 5% CO_2_. Afterwards, 0.5 mg/mL solution was removed and 100 µL/well of the lysis buffer (8 mM HCl + 0.5% NP-40 in DMSO) were added. The absorbance of each sample was measured at 575 nm using the Synergy H1 fluorescence plate reader (Biotek, Bad Friedrichshall, Germany).

### 2.12. In Vitro Evaluation of DPP-IV and ACE Inhibitory Activity

Peptide IAVP (1 mg/mL) was tested as previously described [4,18,19] evaluating its DPP-IV and ACE inhibitory activity, respectively. For the evaluation of in vitro ACE inhibitory activity, a biochemical assay was used (further details are reported in supplementary materials).

### 2.13. Statistical Analysis

The entire data set was checked for normal distribution by D’Agostino and Pearson test. Since they were all normally disturbed with *p*-values < 0.05, we proceeded with statistical analyses by one-way ANOVA followed by Tukey’s post-hoc tests using Graphpad Prism 9 (Graphpad, La Jolla, CA, USA). Values were expressed as means ± s.d. of three independent experiments and each experiment was performed in triplicate; *p*-values < 0.05 were considered to be significant.

## 3. Results

### 3.1. Supramolecular Organization of Peptide Nanostructures

To gain insight into the nanostructure organization of fAPs, we used atomic force microscopy (AFM) morphological analysis (Figure 1B). Before the deposition on fresh mica, each peptide solution (5 μL) was diluted to a final concentration of 0.01% w/v (see Section 2 for further details). The fAP-Soy1 yielded short single nanofibers with 10.02 ± 1.8 and 2.4 ± 0.4, respectively, in width and height, similarly to other reported functionalized RADA16 peptides [20,21,22]. In contrast, fAP-Lup1 did not form nanofibers, but instead led to the formation of sparse round aggregates; this is probably due to the presence of aromatic and aliphatic amino acids within the food bioactive motif. It was, therefore, co-assembled with a pure RADA16 that on its own self-assembles into long nanofibers (Figure S2) [23]. Co-assembly was achieved by mixing solutions of the fAP-Lup1 (1% w/v) and RADA16 (1% w/v). After co-assembly, mixed solutions (namely cAP-Lup1) led to the formation of longer and much more numerous nanofibers if compared to individual fAP-Lup1 solution. The same was observed by mixing solutions of fAP-Soy1 and RADA16 (namely cAP-Soy1). Morphometrical analysis showed that fibers of cAPs ranged from 10 ± 2.5 to 11.65 ± 1.6 nm and 2.1 ± 0.2 to 2.5 ± 0.6 nm, respectively, in width and height.

Next, to investigate the supramolecular arrangement of fAP and cAP peptides, we performed Circular Dichroism (CD), Thioflavin-T (ThT)-binding assay, and Fourier Transform Infrared Spectroscopy (FT-IR). 

**Figure 2:**
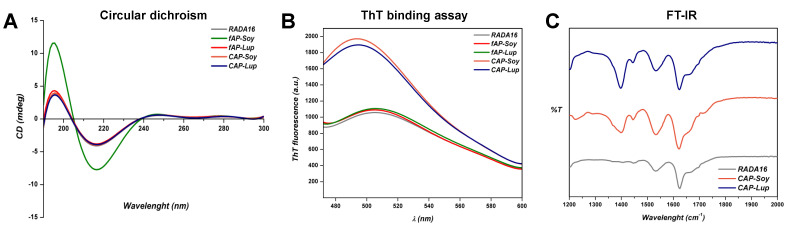
Supramolecular organization of fAPs and cAPs peptide solutions. (**A**) Circular Dichroism (CD) spectra of fAPs and cAPs suggesting the presence of β-sheet secondary structures; (**B**) ThT-binding assay of fAPs and cAPs hydrogels showing typical amyloid emission signals (centered at ~500 nm) that increase in co-assembled fAPs with a pure RADA16; (**C**) FT-IR spectra of fAPs and cAPs hydrogels with characteristic β-sheet peaks in the amide I and amide II regions.

To get an insight on the secondary structures of fAP and cAP assemblies, CD spectra were recorded in the far UV region of 180–300 nm (Figure 2A). All tested peptides showed a CD pattern comprising a negative peak at 215 nm and a positive peak at 195 nm, characteristic of β-sheet secondary structures.

Since the β-sheet structure is characteristic of amyloid-like fibers, typical of SAP molecules, we evaluated this behavior on fAP and cAP peptides using the thioflavin-T (ThT) binding assay [24] (Figure 2B). The fAP samples exhibited a fluorescence intensity peaked at 500 nm comparable to those of RADA16 wild type, ascribable to amyloidogenic structure emissions. Instead, co-assembled fAPs with a pure RADA16 showed high fluorescence levels, probably due to the conformational changes after mixing solutions, thus establishing their strong amyloid-like nature rich in β-sheet structures.

In order to further explore the secondary structure arrangement of both cAPs in solution, we carried out FT-IR spectroscopy tests (Figure 2C). FT-IR spectra of both cAP peptides exhibited a sharp amide I band at 1623 cm^−1^, indicating predominantly β-sheet features. The band at 1,532 cm^−1^ in the amide II region also confirmed the β-sheet aggregation of cAPs, in agreement with the RADA16 spectrum. In addition, both cAP-Soy1 and cAP-Lup1 showed one broad peak at 1400 cm^−1^, which is ascribable to the ring stretching of proline and phenylalanine [25] present in the food bioactive motifs.

Altogether, these data confirmed the self-aggregation of all tested fAPs and cAPs into β-sheets secondary structures, with a characteristic amyloid-like fibers structure. Further, both CD and FT-IR data suggested that co-assembly by mixing solutions of the fAP-Lup1 and fAP-Soy1 with pure RADA16 does not impair the β-sheet formation but, on the contrary, promotes the formation of ordered β-sheet-based nanostructures.

### 3.2. Mechanical Properties of Peptide Nanostructures

**Figure 3:**
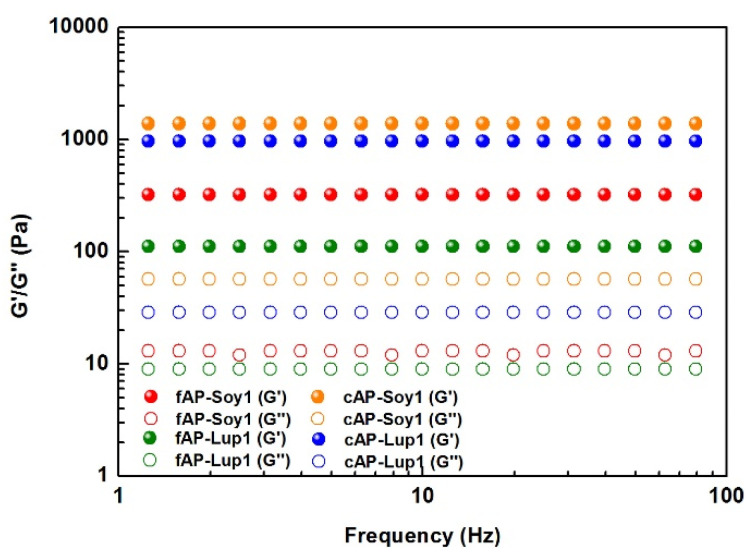
Rheological studies to evaluate the mechanical properties of fAP and cAP hydrogels. Frequency-dependent oscillatory rheology (0.1–100 Hz) of all assembled peptides (1% w/v) highlighted the typical profile of soft hydrogels, featuring a predominant solid-elastic behavior (G’, full dots) as compared to the viscous component (G’’, empty dots).

After confirming the formation of stable β-sheet nanostructures of both fAP and cAPs hydrogels, biomechanical properties of each peptide solution were analyzed via shear-rate rheology (Figure 3). Usually, for β-sheet rich peptide nanofibers the growing presence of such structures leads to formation of an entangled fibrous network, that provides increased G’ values [26]. Hence, the progression of G’ and its comparison with G’’ of all pre-assembled fAP and cAPs solutions were monitored via frequency sweep test (0.1–100 Hz, 1% strain) to evaluate if they showed an elastic (i.e., G’ > G’’, tan δ <1) or viscous (i.e., G’ < G’’, tan δ <1) profile. Though G’ and G’’ remained relatively constant along the tested frequency range, only co-assembled peptide hydrogels displayed significantly increased G’ values (1379 Pa for cAP-Soy1 and 958 Pa for cAP-Lup1). Such values of G’ are probably due to higher organization of cAPs given by their β-sheets-like packed nature seen in ThT experiments, and by nanofiber morphologies shown in AFM analysis.

### 3.3. Biological Evaluation of Co-Assembling Peptide Nanostructures 

**Figure 4:**
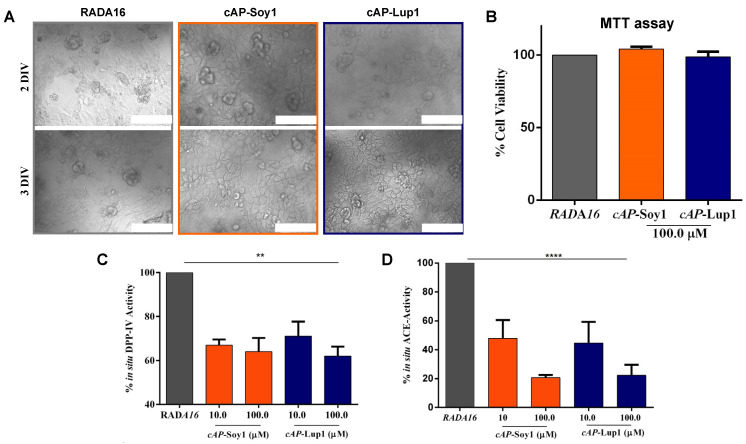
Biological evaluation of co-assembling peptide nanostructures. (**A**) Caco-2 cells cultured over assembled cAP hydrogels (scale bar 100 µm). (**B**) Effects of cAP-Soy1 and cAP-Lup1 on the Caco-2 cells viability. (**C**) Effects of cAP-Soy1 and cAP-Lup1 (10.0 and 100.0 µM) on DPP-IV and (**D**) ACE activities expressed by Caco-2 cells, respectively. Statistically analysis was carried out using one-way ANOVA followed by Tukey’s post-hoc test. (**) *p* < 0.01, (****) *p* < 0.0001. All data sets were statically different vs. RADA16, whereas no statistical significance was observed between cAP-Soy1 and cAP-Lup1 groups in both DPP-IV and ACE assays, respectively.

The potential of cAP-Soy1 and cAP-Lup1 as substrates for human intestinal Caco-2 cells was tested in vitro. A total of 5 × 10^4^/well Caco-2 cells were seeded directly on the top surface of the cAP-Soy1 and cAP-Lup1 hydrogels (see Section 2 for further details). Cells were cultured for 2 and 3 days in vitro (DIV) in order to evaluate the ability of both peptides to act as cell culture scaffolds. As shown in Figure 4A, Caco-2 cells cultured over assembled cAP hydrogels showed spread and fusiform shapes without significant morphological changes compared to Caco-2 cells cultured on RADA16 hydrogel alone. No cytotoxicity effects were observed after 3 DIV by using MTT assay (Figure 4B).

To assess the potential of cAP-Soy1 and cAP-Lup1 as substrates to modulate the DPP-IV and ACE, activity was tested in situ on human intestinal Caco-2 cells. Notably, this cellular system is an interesting survey of membrane peptidases and among these, DPP-IV and ACE stand out [27]. Based on this consideration, our group has already developed, optimized, and validated dedicated cell-based assays in order to measure both DPP-IV [1] and ACE [8,28] activity on Caco-2 cells. 

In order to control the density of food biological signals and therefore attain enhanced bioactivity, thus avoiding the over-expression of the food bioactive motifs, we tested the cAP-based substrates at 10 and 100 μM, respectively.

We observed that cAP-Soy1 reduced the DPP-IV activity by 33.1 ± 2.6% and 36.1 ± 6.2% at 10 and 100 μM, respectively, whereas, cAP-Lup1 inhibited the same enzyme by 29.0 ± 6.5% (10 μM) and 38.0 ± 4.3% (100 μM) (Figure 4C). In addition, to assess the multifunctional properties of cAPs, ACE-inhibitory activity was investigated. Our findings indicate that cAP-Soy1 (10 and 100 μM) inhibits the ACE activity by 52.2 ± 12.7% and 79.5 ± 1.8%, whereas cAP-Lup1 by 55.4 ± 14.5% and 77.8 ± 7.2% at 10 and 100 μM, respectively (Figure 4D).

Both the active motives, Soy1 and Lup1, are able to across the intestinal barrier and during the trans-epithelial transport both peptides are metabolized by the intestinal peptidases located on the apical side of the Caco-2 cells, producing some break-down fragments. Notably, Soy1 is partially degraded in three small peptides AVPTGVA, IAVPT, and IAVP [29], whereas Lup1 is cleaved in two peptides TFPGSAED and LTFPG [28]. In detail, the analysis of the Soy1 sequence using BIOPEP suggests that both AVP and IA are known ACE inhibitor peptides from casein and soybean hydrolysates, respectively, whereas the sequences IA, AV, VP are known as DPP-IV inhibitors, respectively. Hence, experiments were performed for evaluating the ability of IAVP to inhibit both the ACE and DPP-IV activity, respectively. Results suggest that IAVP reduces the in vitro ACE activity by 20.1 ± 0.5% at 1 mg/mL, whereas it is totally ineffective on DPP-IV. Similarly, it has been demonstrated that LTFPG from Lup1 maintains its ACE inhibitory activity losing that against DPP-IV [28]. 

Based on these results and considering that both cAP-Soy1 and cAP-Lup1 are more active as ACE than DPP-IV inhibitors, it is feasible to conclude that when both functionalized cAPs are in contact with the Caco-2 cells, their intrinsic bioactivity is modulated by the cellular system to whom they come into contact. Indeed, their cleavable nature makes smart functionalized cAPs, which may expose a combination of active peptides confirming the cAPs dynamic multifunctional behavior.

## 4. Discussion

We described here a feasible strategy to control and boost up the biological activities of synthetic analogues of two food bioactive peptides derived from the hydrolysis of soybean glycinin and lupin β-conglutin. This was addressed by extending the RADA16 self-assembling peptide sequence at the N-terminus with the two food-derived synthetic analogues. Adopting atomic force microscopy, spectroscopic and rheological techniques, we carried out a thorough investigation of their nanostructures, gelling properties, and biomechanical features. Furthermore, we showed that by co-assembling pure RADA16 with fAP-Soy1 and fAP-Lup1 (namely cAPs), as an approach to control the density of biological signals (i.e., IAVPTGVA and LTFPGSAED), it is possible to achieve greater bioactivity as DPP-IV and ACE inhibitors in Caco-2 cells. 

We previously reported that the non-covalent encapsulation of Soy1 and Lup1 plain solutions within the pre-formed nano-fibrillar hydrogel of RADA16 provides not only higher resistance towards the proteases but also a higher bioavailability of such food bioactive peptides. Furthermore, when these peptides were entrapped within the RADA16 hydrogel, they were released slowly allowing their interaction with the DPP-IV and ACE catalytic sites, suggesting that this strategy could be a viable platform for targeting metabolic diseases. 

However, the main limitation of this strategy is that we needed to encapsulate more than 100 μM of both Soy1 and Lup1 to have a positive effect on DPP-IV and ACE, and then that the two peptides were released with different trends over time; in fact, while Lup1 was regularly delivered by the RADA16 hydrogel during the 360 min of controlled release tests, Soy1 was immediately released within 60–180 min.

Instead, in this work by directly incorporating the synthetic analogues Lup1 and Soy1 sequences to the RADA16 sequence, through a three-glycines spacer to avoid interference between the bioactive motifs and the self-assembling backbone, we tried to bypass the previously reported constraints to improve their stability, bioavailability, and propensity to form bioactive functional hydrogels; as such, Lup1 and Soy1 remain localized and intrinsically exposed in the gelling nanostructures to interact intimately with the cells.

From a morphological point of view, we showed that fAP-Soy1 produces short single nanofibers, and conversely, fAP-Lup1 leads to the formation of round aggregates. Therefore, to ensure a correct nanofibers formation (similar to those of other functionalized RADA16 peptides), and to control the density of the food biological signals and thus obtain a greater bioactivity, avoiding their overexpression (which can be detrimental), we co-assembled fAP-Soy1 and fAP-Lup1 with a pure RADA16, thus leading to the formation of longer and much more numerous nanofibers. Further, CD and FT-IR data suggested that co-assembly by mixing solutions of the fAP-Lup1 and fAP-Soy1 with pure RADA16 does not impair the β-sheet formation (typical of RADA16-based hydrogels) but, on the contrary, promotes the formation of ordered β-sheet-based nanostructures. Moreover, rheological data revealed that co-assembled peptide hydrogels displayed significantly increased G’ values, thus indicating the formation of stiffer and robust co-assembled functionalized hydrogels not only compared to the fAP-Soy1 and fAP-Lup1 samples, but also those previously reported in which Lup1 and Soy1 were entrapped within the pre-formed RADA16 hydrogel.

Further, we have previously demonstrated that both plain solutions of bioactive motives displayed an interesting multifunctional behavior inhibiting the in vitro (IC_50_ of 106 and 228 µM) and cellular (IC_50_ of 223 and 208 µM) DPP-IV activity measured on purified enzymes and on that expressed by Caco-2 cells, respectively [4]. Comparing the IC_50_ values, it appears clear that Soy1 is more active than Lup1 in the biochemical condition, whereas when the same peptides were tested in a cell-based environment, both peptides displayed similar DPP-IV inhibitory potencies. This difference was explained demonstrating that Soy1 is more suitable for the metabolic activity of peptidase expressed by the apical side of human intestinal Caco-2 cells than Lup1 [28]. Indeed, Soy1 (IAVPTGVA) is metabolized in three breakdown fragments (AVPTGVA, IAVP, and IAV) [29], whereas Lup1 (LTFPGSDAD) is degraded only in one metabolite (LTFPG) [28]. In addition, both peptides reduce the ACE activity expressed by Caco-2 cells with a dose–response trend and comparable IC_50_ values, which are equal to 14.7 and 13.7 µM, respectively.

In light of these considerations, our results confirm the multifunctional behavior of both cAP-Soy1 and cAP-Lup1. Indeed, we demonstrated that both intrinsically bioactive hydrogels reduce the ACE and DPP-IV activities, respectively. In more detail, we tested both cAP-Soy1 and cAP-Lup1 at the fixed concentrations of 100 and 10 µM, since these concentrations represent a good compromise with the biological activity on both DPP-IV and ACE enzymes, respectively. In addition, also from a biological point of view the strategy presented in this work provides a useful and alternative system to the encapsulation of bioactive peptides within the RADA16 nanofibers for improving their stability and activity. 

## 5. Conclusions

In conclusion, we synthesized and characterized a nanofiber-based SAP functionalized with two food bioactive peptides derived from the hydrolysis of soybean glycinin and lupin β-conglutin as a viable strategy for controlling and improving their nanostructures, biomechanics, as well as to control the density of food biological signals and therefore attain enhanced bioactivity. 

The nanofiber morphology and multivalent presentation of IAVPTGVA and LTFPGSAED peptides as DPP-IV and ACE inhibitors in vitro on Caco-2 human intestinal cells could offer a future targeting strategy for the prevention of metabolic syndrome through their consumption as edible nanonutraceuticals. 

Future studies will focus on the in vivo therapeutic effect of such nanonutraceuticals to inhibit DPP-IV and ACE and test their behavior to modulate the cholesterol metabolism pathway for targeting the metabolic diseases. 

## Data Availability

Data is contained within the article or supplementary material.

## Supplementary Materials

The following are available online at https://www.mdpi.com/article/10.3390/biomedicines10020330/s1, Figure S1. HPLC and LCMS spectra of fAP-Soy1 and fAP-Lup1; Figure S2. Atomic force microscopy images of pure RADA16.
