# Supplementary material for: Gel-Forming of Self-Assembling Peptides Functionalized with Food Bioactive Motifs Modulate DPP-IV and ACE Inhibitory Activity in Human Intestinal Caco-2 Cells
Source: Biomedicines. 2022 Jan 31;10(2):330. doi: 10.3390/biomedicines10020330 (PMC8869507; doi:10.3390/biomedicines10020330)
Supplement: Supplementary file 1 [file biomedicines-10-00330-s001.zip › biomedicines-1555380-supplementary.pdf]

biomedicines

MDPI

Supplementary Materials for

Gel-forming of self-assembling peptides functionalized with food bioactive motifs modulate DPP-IV and ACE inhibitory activity in human intestinal Caco-2 cells

Raffaele Pugliese[1,*], Martina Bartolomei[2], Carlotta Bollati[2], Giovanna Boschin[2], Anna Arnoldi[2], and Carmen Lammi[2,*]

In vitro evaluation of ACE inhibition activity.

ACE-inhibition activity was tested as already described (Boschin, Scigliuolo, Resta, & Arnoldi, 2014a, 2014b) evaluating hippuric acid (HA) formation from hippuryl-histidyl-leucine (HHL, Sigma Aldrich), as mimic substrate for angiotensin I. 100 µL of 2.5 mM HHL in 100 mM tris-HCOOH, 300 mM NaCl pH 8.3 (buffer 1) was mixed with 30 µL of peptide solution in buffer 1. Six different concentrations of sample were tested and each solution was tested twice. Samples were preincubated at 37 °C for 15 min, then 15 µL of ACE solution (from porcine kidney, Sigma Aldrich), in 100 mM tris-HCOOH, 300 nM NaCl, 10 µM ZnCl2, pH 8.3, were added. Samples were incubated at 37 °C, and after 60 min 125 µL of 0.1 M HCl were added. The aqueous solution was extracted twice with 600 µL of ethyl acetate; the solvent was evaporated, then the residue was dissolved in 500 µL of buffer 1. HPLC analyses were performed with a HPLC 1200 Series (Agilent Technologies, Santa Clara, US) equipped with an autosampler using and a column Lichrospher 100 C18 (4.6 × 250 mm, 5 µm; Grace, Italy). The analytical parameters were: flow rate, 0.5 mL/min; detector, λ 228 nm; mobile phase, water and MeCN, gradient elution from 5 to 60% MeCN in 10min and 60% MeCN for 2 min, then back to 5% MeCN in 3 min; injection volume, 10 µL; Rt (HA), 4.2 min.

The evaluation of the inhibition of ACE activity was based on the comparison between the concentrations of HA in the presence or absence of the inhibitor (Inhibitor Blank). The phenomenon of autolysis of HHL to give HA was evaluated by a reaction blank, i.e. a sample with the higher inhibitor concentration but without the enzyme. The percentage of ACE inhibition was calculated considering the area of the HA peak with the following formula:

$$\%\,\text{ACE INHIBITION} = \left[\frac{(A_{IB}-A_{N})}{(A_{IB}-A_{RB})}\right] * 100$$

where AIB is the area of HA in the Inhibitor Blank (IB) sample (i.e., sample with enzyme but without inhibitor), AN is the area of HA in the samples containing different inhibitor amounts, and ARB is the area of HA in the Reaction Blank (RB) sample (i.e., sample without enzyme and with inhibitor at the highest concentration). The percentages of ACE inhibition were plotted vs Log10 inhibitor concentrations obtaining a sigmoid curve; IC50 was the inhibitor concentration needed to observe a 50% inhibition of the ACE activity and is expressed as mean value ± standard deviation of three independent assays.

In vitro DPP-IV Activity Assay.

The DPP-IV enzyme was provided by Cayman Chemicals (Michigan, USA), while the DPP-IV substrate (H-Gly-Pro-AMC) was from AnaSpec Inc. (Freemont, CA, USA). The experiments were carried out in triplicate in a half volume 96-well solid plate (white). Each reaction (50 µL) was prepared adding the reagents in a microcentrifuge tube in the

following order: 1 X assay buffer (30 μL) [20 mM Tris-HCl, pH 8.0, containing 100 mM NaCl and 1 mM EDTA], IAVP at final concentrations of 1, 10, 100, 500 and 1000 μg/mL or vehicle (C) (10 μL), and finally the DPP-IV enzyme (10 μL). Subsequently, the samples were mixed and 50 μL of each reaction was transferred in each plate well. Each reaction was started by adding 50 μL of substrate solution to each well and incubated at 37° for 30 minutes. Fluorescence signals deriving from the release of free AMC were measured using a Synergy H1 fluorescence plate reader from BioTek (excitation/emission wavelength 350/465 nm respectively).

fAP-Soy1

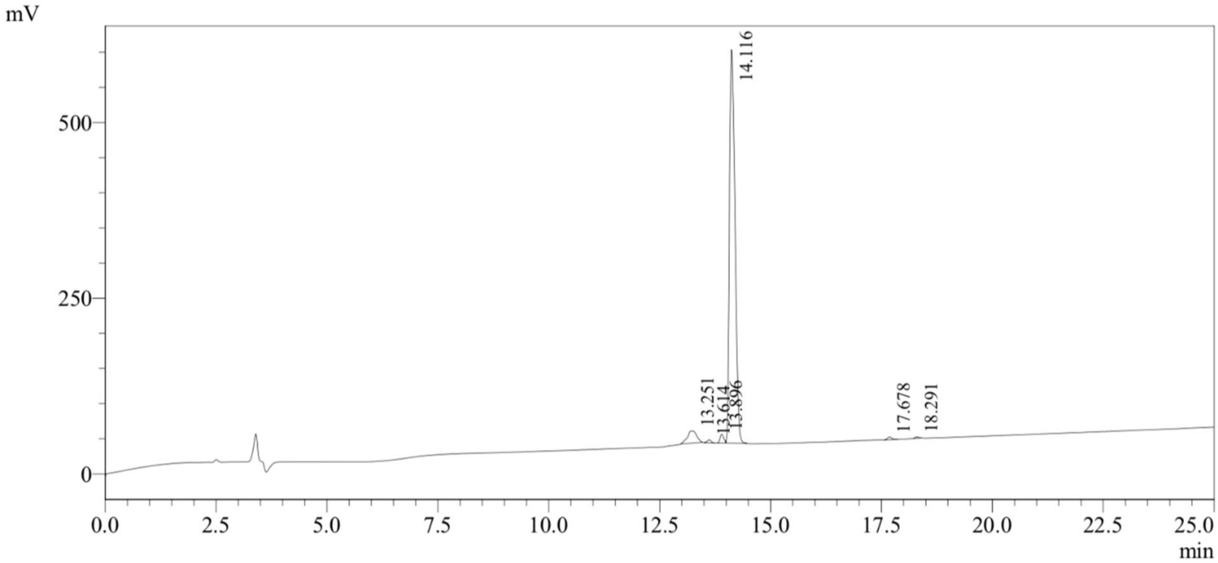

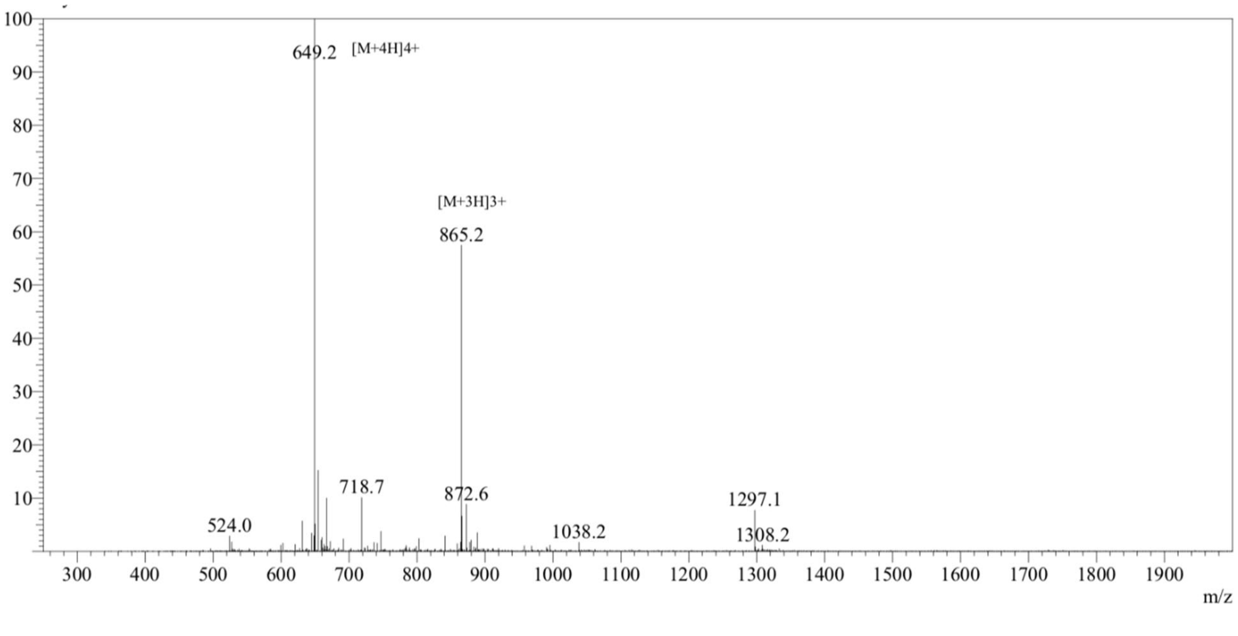

fAP-Lup1

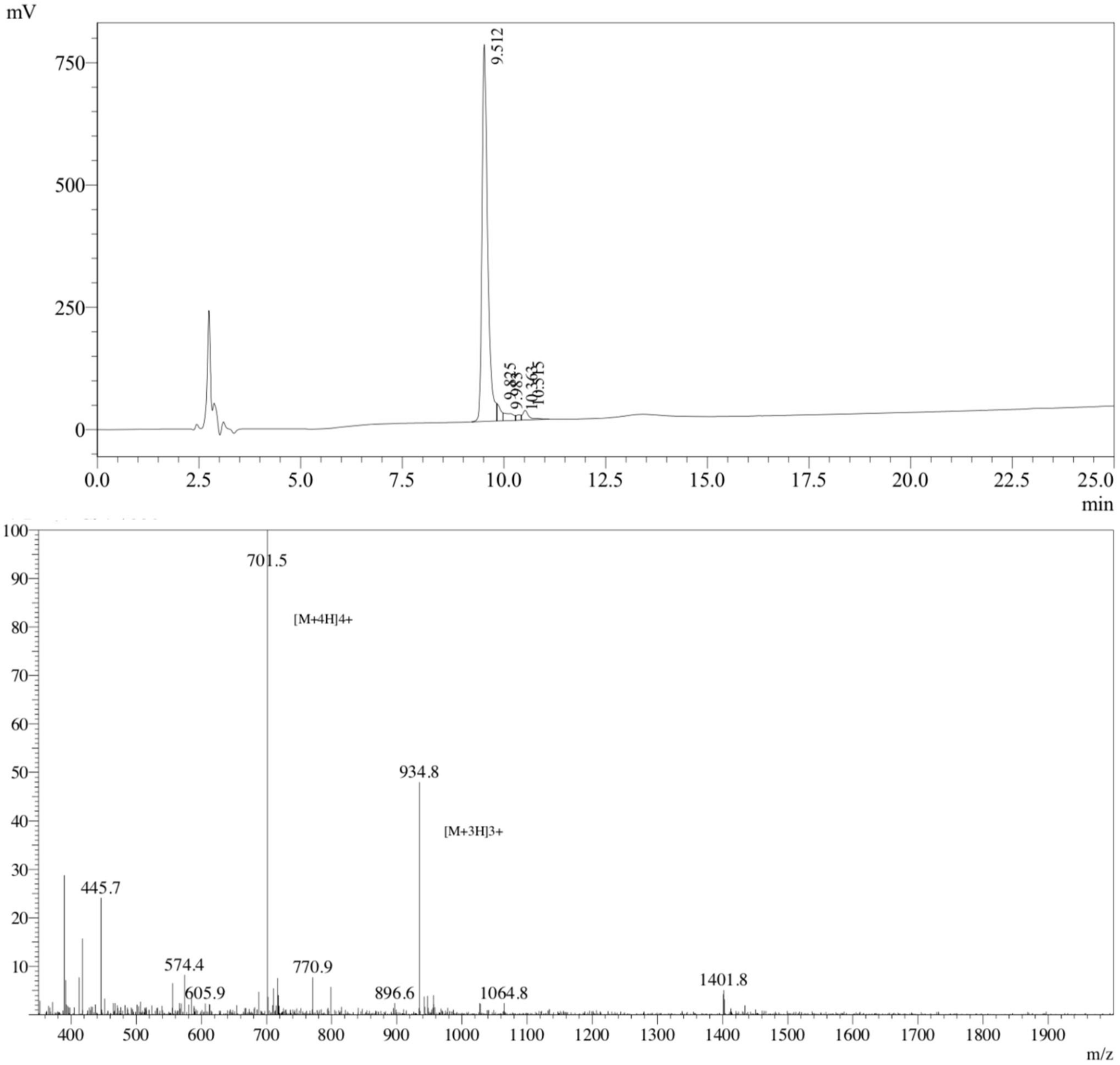

Figure S1. HPLC and LCMS spectra of fAP-Soy1 and fAP-Lup1.

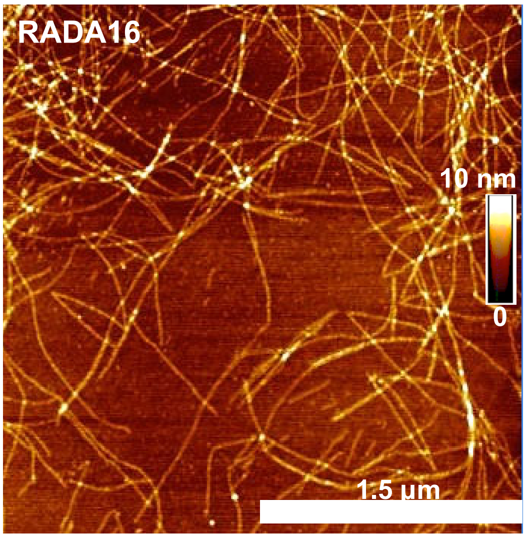

Figure S2. Atomic force microscopy images of pure RADA16.
